# Intraarterial transplantation of human umbilical cord blood mononuclear cells is more efficacious and safer compared with umbilical cord mesenchymal stromal cells in a rodent stroke model

**DOI:** 10.1186/scrt434

**Published:** 2014-04-01

**Authors:** Neha Karlupia, Nathan C Manley, Kameshwar Prasad, Richard Schäfer, Gary K Steinberg

**Affiliations:** 1 Department of Neurosurgery, R281, Stanford School of Medicine, Stanford University, Stanford, CA 94305-5487, USA; 2 Stanford Stroke Center, Stanford University School of Medicine, Stanford University, Stanford, CA 94305, USA; 3 Department of Neurology, All India Institute of medical Sciences, New Delhi 110029, India

## Abstract

**Introduction:**

Stroke is the second leading cause of death worldwide, claims six lives every 60 seconds, and is a leading cause of adult disability across the globe. Tissue plasminogen activator, the only United States Food and Drug Administration (FDA)-approved drug currently available, has a narrow therapeutic time window of less than 5 hours. In the past decade, cells derived from the human umbilical cord (HUC) have emerged as a potential therapeutic alternative for stroke; however, the most effective HUC-derived cell population remains unknown.

**Methods:**

We compared three cell populations derived from the human umbilical cord: cord blood mononuclear cells (cbMNCs); cord blood mesenchymal stromal cells (cbMSCs), a subpopulation of cbMNCs; and cord matrix MSCs (cmMSCs). We characterized these cells *in vitro* with flow cytometry and assessed the cells’ *in vivo* efficacy in a 2-hour transient middle cerebral artery occlusion (MCAo) rat model of stroke. cbMNCs, cbMSCs, and cmMSCs were each transplanted intraarterially at 24 hours after stroke.

**Results:**

A reduction in neurologic deficit and infarct area was observed in all three cell groups; however, this reduction was significantly enhanced in the cbMNC group compared with the cmMSC group. At 2 weeks after stroke, human nuclei-positive cells were present in the ischemic hemispheres of immunocompetent stroke rats in all three cell groups. Significantly decreased expression of rat *brain-derived neurotrophic factor* mRNA was observed in the ischemic hemispheres of all three cell-treated and phosphate-buffered saline (PBS) group animals compared with sham animals, although the decrease was least in cbMNC-treated animals. Significantly decreased expression of rat interleukin *(IL)-2* mRNA and *IL-6* mRNA was seen only in the cbMSC group. Notably, more severe complications (death, eye inflammation) were observed in the cmMSC group compared with the cbMNC and cbMSC groups.

**Conclusions:**

All three tested cell types promoted recovery after stroke, but cbMNCs showed enhanced recovery and fewer complications compared with cmMSCs.

## Introduction

Cells derived from the human umbilical cord (HUC) have been successfully used in the clinic for almost 2 decades [1-4]. Their simple and economic retrieval, enrichment for hematopoietic progenitors, enhanced proliferation rate, expansion potential [5,6], and low incidence of graft-versus-host disease [7,8] makes them a promising cell treatment for several disorders. Although their therapeutic benefits were initially thought to be limited to hematopoietic disorders, several recent studies have shown the potential of these HUC-derived cells to enhance regeneration and tissue repair in various pathological disorders, including neurologic diseases [9-11]. HUC-derived cells have already been used clinically for nonhematopoietic degenerative conditions [12], hereditary ataxia [13], and disorders such as cerebral palsy [14] and spinal cord injury [15], and they are currently being tested for neonatal hypoxic-ischemic encephalopathy (clinicaltrials.gov/ct2/show/NCT00593242).

HUC-derived cells have been used in preclinical stroke studies for more than a decade. Alhough most studies have shown significant functional or histo-pathologic recovery, homing, and differentiation of the grafted cells [16-25], some studies reported on lack of migration or neurologic benefits [26-28] or absence of human nuclei-positive cells despite evidence of functional recovery [29]. In a meta-analysis, we assessed the effects of HUC-derived cells in preclinical stroke studies (included studies, 14). We found a significant reduction in infarct volume with an overall effect of Z = 6.54 (*P <* 0.00001), with a noticeable heterogeneity as well (I^2^ = 87%). Apart from variation in study design, cell dose, or route of transplantation, a possible source of this heterogeneic outcome could be the varying mix of cell populations present in human umbilical cord blood [11] and/or biologic differences of the cord matrix/blood-derived cell populations used in these studies. cbMNCs comprise a heterogeneous mix of immature lymphocytes, monocytes [12,30], hematopoietic [31], endothelial [32], and mesenchymal [33-36] stem/progenitor cells. Moreover, studies report on the presence of very small embryonic-like stem cells [37], embryonic-like cells [38,39], and neural stem/progenitor cells [40-42] at different lineage commitment stages. It is therefore important to understand whether it is the mixture of cord blood cells or a specific stem cell population that is responsible for ameliorating the harmful effects of stroke and hereby to assess the risks of cell therapy.

In this study, we characterized and compared cbMNCs from HUCs with two entities of HUC-derived mesenchymal stromal cells (MSCs): cord blood MSCs (cbMSCs), and cord matrix MSCs (cmMSCs). Whereas cbMSCs are composed of a rare population of multipotent precursors enriched from cbMNCs [35,43,44], cmMSCs are derived from the mucoid connective tissue surrounding cord blood vessels, known as Wharton jelly [45]. These three cell populations were transplanted into stroke rats to assess their potential effects on recovery and their ability to home to the host brain after intraarterial transplantation. Additionally, we analyzed the inflammatory response in the host, as well as the expression of *brain-derived neurotrophic factor (BDNF), epidermal growth factor (EGF), glutathione peroxidase-4 (GPx-4),* and *reelin* mRNA in the ipsilateral hemispheres of the study animals, given their role in neuroprotection and recovery after stroke [46-56].

## Materials and methods

### Transient middle cerebral artery occlusion model and sham surgery

All animal procedures were approved by the Stanford University Administrative Panel on Laboratory Animal Care. Adult male Wistar rats (270 to 300 g) were subjected to middle cerebral artery occlusion (MCAo) by using the 2-hour transient intraluminal filament occlusion model of cerebral ischemia, as described previously [49,57,58]. In brief, rats were anesthetized with 2% isoflurane in a mixture of 30% oxygen and 70% air. After a midline incision, the right common carotid artery (CCA), external carotid artery (ECA), and internal carotid artery (ICA) were exposed. The CCA and ICA were temporarily ligated, and a 4–0 silicone rubber-coated reusable monofilament (Doccol Inc, 40SPRe3756) was inserted into the lumen of the right ICA through a small incision in the ECA. The filament was advanced into the ICA until 18 to 19 mm was inserted from the bifurcation, and the origin of the middle cerebral artery was blocked. Physiological parameters (respiratory rate, core body temperature, and color of mucous membranes) were monitored and recorded throughout the surgical procedure. Two hours after the MCAo, each animal was reanesthetized, and the monofilament was withdrawn to cause reperfusion. All surgery animals were given analgesic treatment with 0.03 mg/kg buprenorphine.

For sham surgery, the ECA was surgically prepared for insertion of the filament, but the 4–0 monofilament was not inserted. The neck incision was closed, and each animal underwent the same postoperative care as the stroke surgery animals.

### Isolation of cbMNCs and culture of MSCs

Cord blood collection was approved by the Institutional Review Board (IRB) and Stem Cell Research Oversight (SCRO) Committee at Stanford University. Informed consent was taken from donor mothers before samples were collected. All six cord-blood samples were processed within 10 hours of collection, and cbMNCs were obtained by using the Ficoll-Paque density gradient method [40,59,60]. Cell viability was tested by using the trypan blue exclusion method. The cbMNCs obtained were used for cbMSC culture, flow-cytometry experiments, transplantation, or cryopreservation. cbMSC cultures were established, as previously described [61], with modifications made in our laboratory. In brief, 60 × 10^6^ cbMNCs were plated per fibronectin-coated six-well plates (Nunc, Thermo Fisher Scientific, Waltham, MA, USA) in proliferation medium comprising MEM-α with glutamax, ribonucleosides, and deoxyribonucleosides (Invitrogen, Grand Island, NY 16000–044), 10% fetal bovine serum (Gibco FBS), 50 n*M* dexamethasone (Sigma-Aldrich, St. Louis, MO D8893), 10 ng/ml epidermal growth factor (Sigma-Aldrich E9644), 10 ng/ml platelet-derived growth factor (R&D Systems, Minneapolis, MN), and 1× penicillin-streptomycin (Invitrogen 15140–122). Plates were placed in a normoxic humidified incubator with 5% CO_2_ and growth medium was replaced after 24 hours of plating and every alternate day thereafter.

On appearance of the first colony of adherent cells, cells were harvested by using 0.25% trypsin-EDTA solution and re-plated in new noncoated culture plates. Passages 5 to 6 were used in all experimental procedures. cmMSCs were obtained commercially from PromoCell (C-12971, Germany) and were expanded by using proliferation medium (C-28010, PromoCell).

### Flow-cytometry analysis

Flow cytometry was used to characterize the cultured cbMSCs and to compare them with cbMNCs and cmMSCs. cbMSCs from passages 5 and 6 were assessed for expression of mesenchymal and hematopoietic lineage markers relative to cbMNCs in successive flow-cytometry experiments by using mouse anti-human CD73 (PE), CD90 (FITC), CD166 (PE), CD34 (FITC), CD133 (APC), CD19 (FITC), CD45 (APC-Cy7), HLA-ABC (FITC), and HLA-DR (FITC). cbMSCs were further compared with cmMSCs and cbMNCs simultaneously by using a multicolor flow-cytometry experiment designed for a total of 27 phenotypic markers (see Additional file 1). cbMSCs and cmMSCs used in this experiment were from passage 6, and cbMNCs used were from the same sample from which the cbMSC culture was established. For comparative immunophenotyping, cbMNCs, cbMSCs, and cmMSCs (75,000 cells per experiment) were washed with phosphate-buffered saline (PBS) containing 0.1% to 1% FBS, and 100-μl aliquots of each were placed in flow-activated cell sorting (FACS) tubes for staining.

Six antibody cocktails (Additional file 1) were used for characterization of the three cell types and were prepared according to their conjugated fluorochromes and their emission ranges. The volume of each antibody used was per manufacturer’s recommendation or as titrated in preliminary experiments. Antibody cocktails were added to each tube and incubated at 4°C for 30 minutes. Stained cells were given three washes at 1,200 rpm for 5 minutes each and then suspended in 500 μl FACS buffer. Simultaneously, compensation controls were prepared for all the markers by using BD-CompBeads (BD Biosciences, San Jose, CA). Flow cytometry was performed on a Becton Dickinson LSR-II (Stanford Shared FACS Facility), and data were analyzed with FlowJo (TreeStar Inc). Gates were set according to the unstained and 7-amino-actinomycin D-stained controls to exclude dead cells for each cell type, and compensation was done by single-color-stained BD-CompBeads. Isotype controls were not used for these experiments, as several past studies pointed to their nonessential [62-64] or potentially misleading results [65,66].

### Transplantation of cbMNCs, cbMSCs, or cmMSCs

Animals were randomly divided into experimental groups: sham surgery (*n* = 12); MCAo + PBS (*n* = 27); MCAo rats infused with 10 × 10^6^ cbMNCs (*n* = 24); 5 × 10^6^ cbMSCs (*n* = 18); 5 × 10^6^ cmMSCs (*n* = 23), and 10 × 10^6^ cmMSCs (*n* = 6). Stroke animals were injected with PBS or cells suspended in PBS 24 hours after stroke through an intracarotid route. To assess the inflammatory reaction after stroke, none of the experimental animals received an immunosuppressant drug.

### Assessment of neurologic deficit

The rotarod, grid-walk, and open-field behavior tests were used to assess motor coordination, sensory motor function, and spontaneous activity of study animals before surgery (MCAo/sham), and at 1, 4, 7, and 14 days after surgery. Baseline readings at 24 hours before surgery were the internal controls for each study animal. All behavior experiments were performed between 9 AM and 2 PM.

In the rotarod performance test rats were placed on a motor-driven cylinder and the length of time they remained on it before falling was recorded [16,67,68]. Animals were trained 3 days before surgery to remain on the rotating spindle (moving at a constant speed of 8 rpm) for 60 seconds. One day before surgery, the trained animals were given a test trial on an accelerating cylinder (with an increasing speed of 4 to 40 rpm within 5 minutes), and latency to their fall was recorded.

The grid-walk test was modified from the test described by Rogers *et al.*[68]. Rats were placed on an elevated 30-mm^2^ grid floor for 1 minute to be acclimated and then placed on the grid floor and recorded for 2 minutes. Total numbers of paired steps as well as total numbers of foot faults for each limb were manually counted from the recorded videos [67]. Percentage foot fault was determined by using the formula: ((Number of foot faults/(number of paired steps + number of foot faults)) × 100).

For the open-field test, animals were individually placed in the center of the arena and were tracked with a video camera connected to a computer. Ethovision XT 5.1 software by Noldus was used to design the paradigm for recording, and animals were recorded for 10 minutes for each trial. Total distance moved by each animal during the 10-minute trial was compared to assess spontaneous activity.

### Assessment of infarct size

On day 14 after stroke, rats were killed with isoflurane and perfused transcardially with 0.1 *M* PBS (pH 7.4) followed by 4% paraformaldehyde (PFA). Brains were extracted and incubated overnight in 10-fold 4% PFA and were saturated with increasing sucrose concentrations (20% followed by 30%) in PBS for 24 to 48 hours. Cryosections (20 μm) were cut serially between +1 to -1 bregma. Seven coronal sections (200 μm apart) per brain were stained by using high-contrast silver staining and scanned at 1,200 dpi [69,70]. Image J software by the National Institutes of Health (NIH) was used to measure the total ipsilateral, contralateral, and infarct areas. Infarct area was calculated relative to the contralateral hemisphere by using the formula: infarct size = 100 × (total contralateral hemisphere area - (total ipsilateral hemisphere area–infarct area)/total contralateral hemisphere area) [17,69].

### Tissue processing and immunohistochemistry

At 2 weeks after stroke, animals were transcardially perfused, and brains were extracted and sectioned at 20-μm postfixation. Immunohistochemistry was performed for localization of human nuclei (HuNu)-positive cells in cell-treated animals and to assess the effect of transplanted cells on immune response by using anti-CD68 and ionized calcium-binding adapter molecule-1 (Iba-1) antibodies.

Immunohistochemistry to localize transplanted human cells was performed [71] by using a monoclonal antibody specific for human nuclei with no cross-reactivity for rat or mouse nuclei (MAB1281, mouse anti-HuNu, Millipore, Billerica, MA). In brief, endogenous peroxidase activity was quenched with H_2_O_2_ (0.3%), and sections were blocked for 40 minutes in tris-buffered saline (TBS) containing 5% normal goat serum (NGS; Vector Laboratories, Burlingame, CA, S-1000). Sections were incubated overnight at 4°C with primary antibody (1:100) and then incubated with biotinylated secondary antibody (goat anti-mouse IgG, Zymed; Vector Laboratories, 1:200) for 2 hours at room temperature followed by ABC (Vectastain Elite; Vector Laboratories) and diaminobenzidine (DAB; Vector Laboratories) developing. Immunohistochemical staining in the contralateral hemisphere was used as an internal control for each animal. Sections from the PBS-group animals were also stained as a negative control for human cells.

A similar procedure was followed with the lysosomal marker CD68 by using mouse monoclonal anti-rat CD68 antibody (sc-59103; Santa Cruz Biotechnology, Inc., Santa Cruz, CA, 1:200).

For Iba-1, immunofluorescence was performed. Primary antibody (rabbit anti-Iba-1; Wako; 1:600) was incubated overnight at 4°C followed by 2 hours of incubation at room temperature with secondary antibody diluted at 1:400 (Alexa Fluor-594 goat anti-rabbit; Invitrogen, Life Technologies, Grand Island, NY-A11037) [71]. Mounting was done by using Vectashield mounting media with DAPI (Vector Laboratories, H-1200), and images were taken by using a Zeiss fluorescence microscope.

### Total RNA isolation, reverse transcription, and quantitative real-time PCR

Total RNA was isolated by using the Qiagen RNeasy Mini Kit and following manufacturer’s instructions. In brief, rats were killed at day 14, and the brains were extracted. About 3 mm^3^ (approximately 30 mg) of tissue was excised from the ischemic hemisphere, 2 to -1.5 bregma, and just medial to the infarcted region of cell-treated and PBS-group animals. A similarly representative brain region was excised from the ipsilateral hemisphere of sham animals. Reverse transcriptase reactions were performed with the Bio-Rad iScript cDNA synthesis kit by using 1 μg of total RNA.

To analyze levels of mRNA, qPCR was performed on the Bio-Rad CFX96 Real-Time Detection System by using Taqman gene-expression assays (Inventoried/Cat. 4331182; Applied Biosystems, Carlsbad, CA, USA) according to the manufacturer’s instructions. Reference genes were rat- and human-specific *glyceraldehyde-3-phosphate dehydrogenase* (*GAPDH*) (Rn01775763_g1; Hs03929097_g1*). Target genes were rat-specific *brain-derived neurotrophic factor* (*BDNF*) (Rn02531967_m1); g*lutathione peroxidase-4* (*GPx-4*) (Rn00820816_g1); *epidermal growth factor* (*EGF*) (Rn00563336_m1); *reelin* (Rn00589609_m1); *interleukin 1beta* (*IL-1β*) (Rn00580432_m1); *tumor necrosis factor alpha* (*TNF-A*) (Rn01525859_g1); rat- and human-specific *interleukin* (*IL*)*-2* (Rn00587673_m1, Hs00174114_m1); *IL-6* (Rn01410330_ml, Hs00985639_m1); and *IL-10* (Rn0053409_m1, Hs00961622_m1).

The threshold cycles (Ct) for the target gene and internal control were determined for each sample, which underwent qPCR in parallel duplicate runs. Expression of target genes was normalized to the housekeeping gene *GAPDH* and is displayed as 2^-ΔCT^.

### Assessment of adverse effects and mortality rate

Appearance of adverse effects during/within 24 hours and 24 hours after cell administration was recorded. The total of dead animals in each group was counted separately for time points <24 hours and >24 hours after transplantation. The mortality rate was calculated as percentage of animals that died of total animals in each group. Statistical significance for mortality was calculated relative to PBS group.

### Statistics

Statistical analyses of data were performed with GraphPad Prism 5 software (San Diego, CA, USA). One-way analysis of variance (ANOVA) with Tukey *post hoc* test was used for percentage of infarct area, and two-way ANOVA (with Bonferroni *post hoc* test) was used for behavioral analysis. The *t* test with Bonferroni multiple-comparison adjustment was used for qPCR analysis. All neurologic, histopathologic, and gene-analysis data are presented as mean ± SEM. For flow-cytometry data analysis, the two-tailed *t* test with Welch correction was used, and data are presented as mean ± SD. For mortality data analysis, the Fisher Exact test was used with two-tailed *P* values. Significance was set at *P <* 0.05.

## Results

### Culture of cbMSCs and comparative immunophenotyping of cbMNCs, cbMSCs, and cmMSCs with flow cytometry

**Figure 1:**
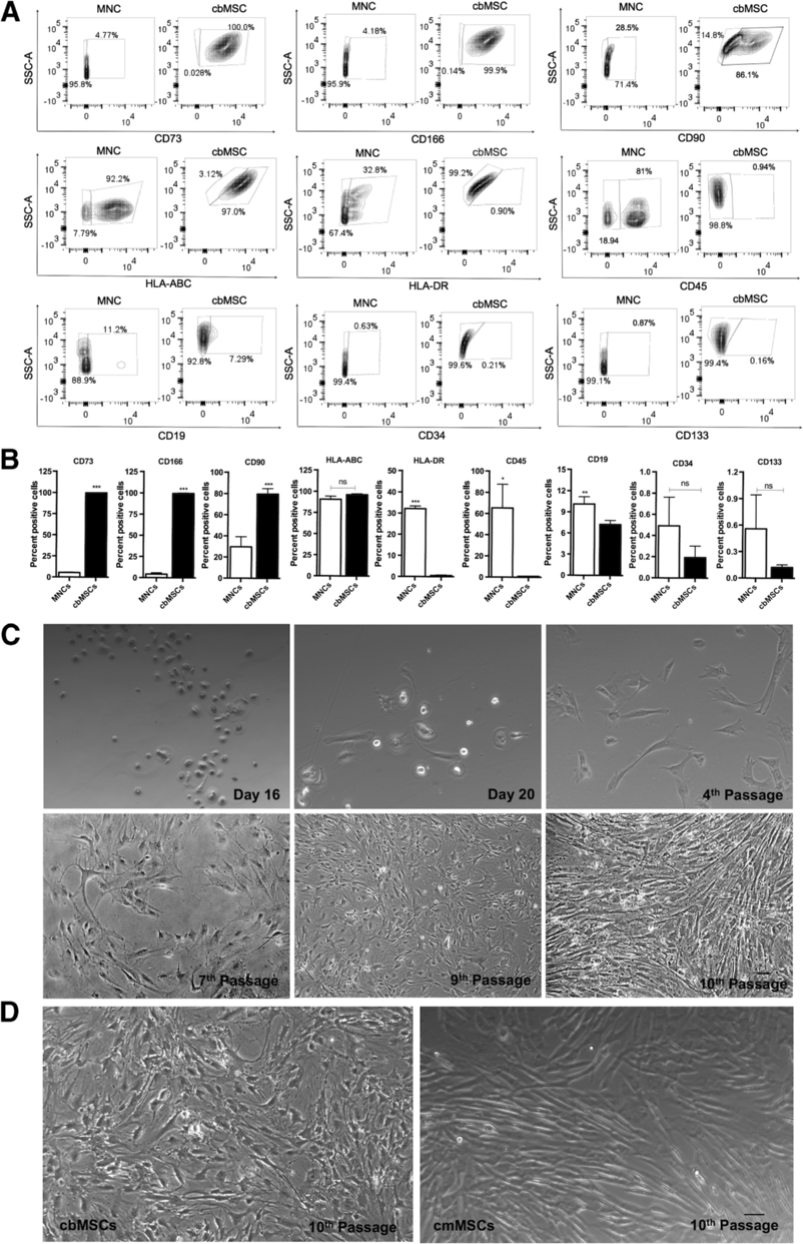
**Immunophenotypic characterization and expansion of cultured cord-blood MSCs.** Culturing cbMSCs from cbMNCs resulted in enrichment of CD73^+^CD166^+^CD90^+^CD45^-^CD34^-^HLA-DR^-^ cells closely resembling the phenotypes of MSC proposed by the International Society for Cellular Therapy (ISCT), 2006 [72]. Representative flow-cytometry contour plots are shown to illustrate expression of individual phenotypic markers in cbMNCs and cbMSCs **(A)**. In each plot, percentage of cells positive for a given marker is shown on the right, and percentage of cells negative for the same marker is shown on the left. Relative to cbMNCs, cbMSCs had enriched expression of CD73, CD166 (*P* < 0.0001), CD90 (*P <* 0.0004), and decreased expression of HLA-DR (*P <* 0.0001), CD45 (*P <* 0.01), and CD19 (*P <* 0.005). cbMSCs also had relatively higher expression of HLA-ABC and lower expression of CD34 and CD133, although the difference was not significant **(B)**. Spindle-shaped, fibroblast-like, flat, adherent cells are shown across various passages of cbMSC culture (magnification 10×) **(C)**. Comparative morphologic representation of 10th passage cbMSCs and commercially obtained cmMSCs is shown in **(D)**. Student *t* test, *n =* 4 for each group, (**) is *P <* 0.01, (***) is *P <* 0.001, and (ns) is nonsignificant. Data represent means ± SD.

Attempts to isolate MSCs from six cord-blood samples resulted in two colonies of adherent cells that showed a flat, spindle-shaped, fibroblastoid phenotype (Figure 1). The first colony from these two cord blood samples was visible at days 16 and 18, respectively. Cultured cbMSCs were assessed at passages 5 and 6 for the expression of surface antigens reportedly expressed on MSCs [72], and were found positive for CD90, CD73, CD166, and showed negligible expression of CD45 and HLA-DR. Relative to cbMNCs, enriched cbMSCs showed higher expression of CD73, CD166 (*P* < 0.0001), CD90 (*P* < 0.0004) and decreased expression of HLA-DR (*P* < 0.0001), CD45 (*P* < 0.01), and CD19 (*P* < 0.005) (Figure 1A,B).

Enriched cbMSCs showed a trend toward higher expression of HLA-ABC and lower expression of CD34 and CD133. CbMSCs were compared with cbMNCs and commercially obtained matrix-derived cmMSCs by using multicolor flow cytometry (Table 1; see Additional files 2, 3). cbMNCs were positive for MHC I, MHC II, dendritic, hematopoietic, leukocytic, endothelial, lymphocytic, and myeloid cell markers (CD56, CD14, CD45, LIN1, CD33), but, like cb/cmMSCs, showed very low expression of primitive hematopoietic progenitors (CD34, CD133). Both cbMSCs and cmMSCs were highly positive for CD44, CD117, CD73, CD166, CD184, CD59, CD123, and CD210, and showed very low expression of HLA-DR, CD34, CD33, CD133, and CD45. Apart from the expression of MSC lineage markers, expression of CD117 (stem cell factor), CD184 (CXCR4 receptor), CD106 (VCAM-1 molecule), CD59 (inhibitor of membrane attack complex formation), and lack of Lin1^+^ cells (only in cbMSCs), mainly distinguished MSCs from cbMNCs (Table 1 and Additional files 2, 3).

**Table 1:** Percentage expression of phenotypic cell markers in cbMSCs, cmMSCs, and cbMNCs

**Marker**	**cbMSC**	**cmMSC**	**cbMNC**	**Function of marker**
CD7	0.407	5.4	62.51	Marker for thymocytes and mature T cells
CD4	5.88	68.0	26.77	Marker for T-helper cells, regulatory T cells, monocytes
CD25	91.2	71.3	71.8	Part of IL-2 receptor present on activated T cells/B cells
CD3	36.3	4.33	31.3	T-cell coreceptor and marker for mature T cells
CD8	0.123	0.277	21.1	Coreceptor for T-cell receptor and binds specifically to major histocompatibility complex (MHC) I
CD44	100	99.7	81.7	Marker for effector memory T cells, lymphocyte activation, receptor for hyaluronic acid
HLA-ABC	96.9	98.8	92.4	MHC Class I
CD90	73.5	99.8	36.5	Marker for mesenchymal stem cells, hematopoietic stem cells (HSCs), and fibroblasts
CD56	1.28	80.8	11.3	Neuronal cell-adhesion molecule, plays role in synaptic plasticity and neurite outgrowth
CD210	91.8	96.5	0.544	IL-10 receptor on T cells/B cells, natural killer (NK) cells, and monocytes
CD45	0.99	0.82	87.7	Leukocyte common antigen
CD14	0.404	20.3	12.4	Monocyte differentiation antigen
CD34	0.124	1.57	0.80	Marker for primitive hematopoietic and endothelial stem cells
CD133	0.11	0.899	0.897	Prominin-1, specific for hematopoietic stem cells, endothelial progenitors, neuronal and glial stem cells
CD117	99.5	92.9	2.2	Stem cell growth-factor receptor, expressed on HSCs, multipotent progenitors, common myeloid progenitors
CD73	100	99.5	5.75	Lymphocyte differentiation marker
CD123	91.8	48.8	1.81	IL-3 receptor on pluripotent progenitors
CD16	31.5	72	81.9	Marker for NK cells, monocytes, and macrophages
CD166	100	99.8	3.38	Activated leukocyte cell-adhesion molecule
Lin1	0.46	31.6	87.2	Lineage cocktail 1 (CD3, 14, 16, 19, 20, 56)
CD59	99.8	99.3	7.14	Complement regulatory protein, regulates complement-mediated cell lysis by formation of membrane-attack complex
CD106	16.21	23.0	3.54	Vascular cell-adhesion molecule (VCAM)-1
CD184	100	75.6	64.4	Chemokine (C-X-C motif) receptor-4 specific for stromal-derived factor-1
HLA-DR	0.434	0.312	31.6	MHC Class II
CD19	6.89	0.399	9.27	Marker for follicular dendritic cells and B cells
CD33	1.26	0.313	27.3	Marker for myeloid-lineage cells

Represents comparative percentage expression of various markers assessed with flow cytometry by using Becton Dickinson LSR II (Stanford Shared FACS facility).

Although cmMSCs expressed typical MSC markers, these cells also exhibited expression of certain markers (Lin1^+^ 31.6% compared with 0.46% for cbMSCs; CD56, 80% compared with 1.28% for cbMSCs; CD4, 68% compared with 5.8% for cbMSCs and CD14, 20% compared with 0.404% in cbMSCs), similar to cbMNCs. CD56, expressed on neuronal and NK cells and known to mediate synaptic plasticity, was found highest in cmMSCs (80.8%) compared with 11.3% in cbMNCs and 1.28% in cbMSCs.

### cbMNCs are as effective as cbMSCs or cmMSCs in improving motor coordination

**Figure 2:**
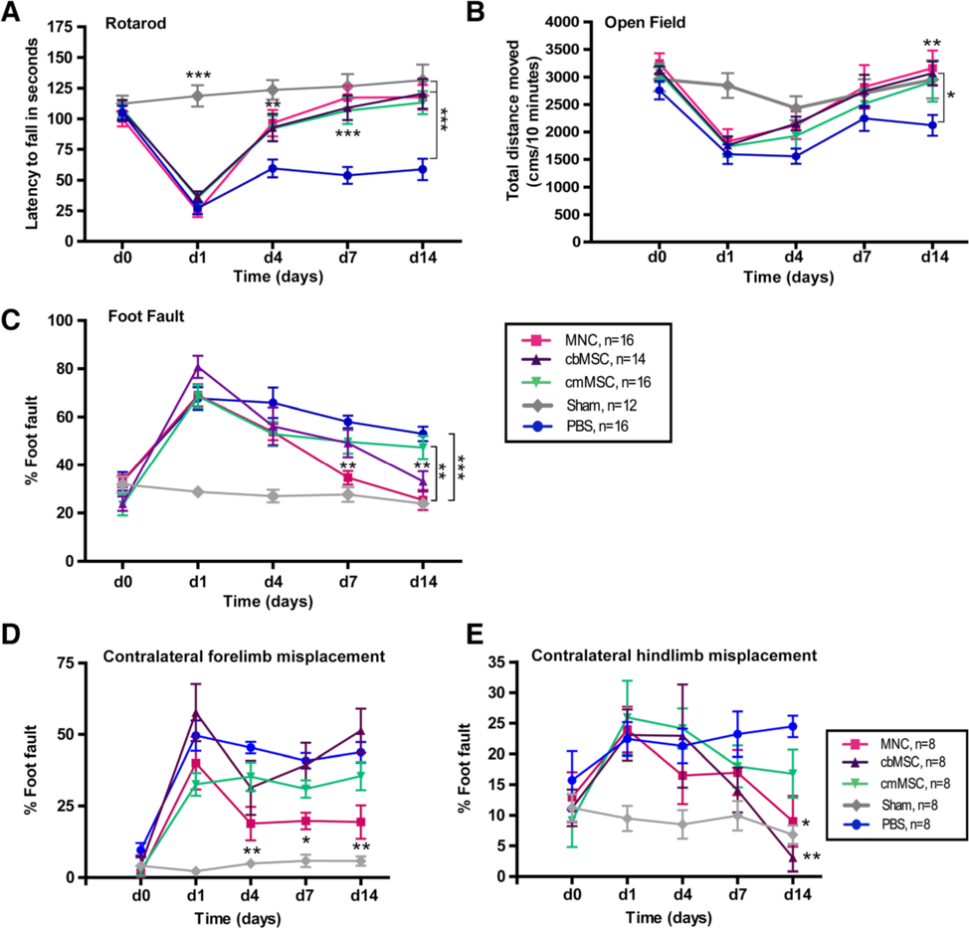
**Reduced neurologic deficits in all three cell-treated stroke animals with enhanced recovery in the cbMNC group.** Neurologic deficits were assessed on rotarod **(A)**, open field **(B)**, and foot-fault **(C)** tests before stroke (d 0) and at d 1, 4, 7, and 14 after stroke. Rotarod motor coordination of all cell-treated animals improved significantly compared with the PBS group. *P <* 0.01 (d4), *P <* 0.001 (d 7, 14) for cbMNC group; *P <* 0.05 (d 4) and *P <* 0.01 (d 7, 14) for cbMSC and cmMSC groups. Significant improvement in open-field spontaneous activity was seen at d 14 in cbMNC- and cbMSC-treated animals, but not in the cmMSC group compared with the PBS group (*P <* 0.01 and *P <* 0.05, respectively). Significant reduction in percentage foot faults was seen in the cbMNC group relative to the PBS group (*P <* 0.01, d 7 and *P <* 0.001, d 14) and the cmMSC group (*P* < 0.05, d 14). cbMSC- but not cmMSC-treated animals showed significant reduction in limb misplacement at d 14 (*P <* 0.01). Relative to the PBS group, only the cbMNC group showed significantly less contralateral forelimb errors **(D)** (*P <* 0.01 at d 4; *P <* 0.05 at d 7, 14); however, both cbMSC and cbMNC groups showed recovery in contralateral hindlimb errors at d 14 **(E)** (*P <* 0.01 and *P <* 0.05, respectively). At d 14, relative to the sham group, no significant difference was seen in contralateral forelimb errors in the cbMNC group and in hindlimb errors in both cbMNC and cbMSC groups **(D, E)**. (*) is *P <* 0.05; (**) is *P <* 0.01, and (***) is *P <*0.001. Values are expressed as mean ± SEM.

Compared with the PBS group, all three cell-group animals showed significant recovery in the rotarod test at day 4 (*P* < 0.01 for MNC and *P* < 0.05 for cbMSC and cmMSC), which continued until day 14 after stroke (*P* < 0.001, cbMNC; *P* < 0.01, cbMSC and cmMSC). At 24 hours after stroke, the latency to fall from the rotating spindle was significantly shorter in MCAo versus sham animals; however, no significant differences were seen in the sham-versus-cell groups or within the three cell-treated groups at days 4, 7 and 14. By 2 weeks, all three cell groups were performing at prestroke baseline levels (Figure 2A).

### cbMNCs and cbMSCs but not cmMSCs promote improvement in spontaneous activity after stroke

MCAo caused a significant decrease in spontaneous activity of stroke rats compared with the sham group at day 1. Improvement in spontaneous activity, although not significant, was seen at day 4 after stroke in cell-treated animals and at day 7 in PBS-group animals. Despite continuous improvement in the spontaneous activity of cell-treated rats from days 4 to 14, significant recovery relative to the PBS group was seen only at day 14 after stroke in the cbMNC and cbMSC groups but not in the cmMSC group. PBS-treated MCAo rats showed increased spontaneous activity at day 7, which showed a deteriorating trend at day 14. No significant difference was seen across the three cell groups (Figure 2B).

### cbMNCs are more effective in reducing sensory-motor deficits than are cbMSCs or cmMSCs

All study animals showed similar limb misplacements in the grid-walking test before stroke, which increased significantly after stroke relative to the sham group. Compared with the PBS group, all cell-treated animals showed a decreasing trend in percentage of foot faults, starting on day 4 (Figure 2C). However, only the cbMNC and cbMSC groups showed significant reductions in limb misplacement at day 14 after stroke (*P* < 0.001 for cbMNCs and *P* < 0.01 for cbMSCs) compared with the PBS group. Only the cbMNC group showed significant reduction in foot faults starting on day 7 (*P* < 0.01) and, by day 14, percentage of foot faults in this group was as low as prestroke baseline values. At day 14, the cbMNC group performed significantly better than the cmMSC group (*P* < 0.05), but there was no significant difference between cbMSC and cmMSC groups.

A subgroup analysis (*n* = 8 per group) showed significant recovery in both contralateral forelimb and hindlimb function of cbMNC-treated animals and only contralateral hindlimb function of cbMSC-group animals compared with PBS-group animals (Figure 2D,E). cbMNC-group animals displayed significantly fewer faults in the contralateral forelimb at day 4 (*P* < 0.01) and at days 7 and 14 (*P* < 0.05), compared with PBS-group animals, (Figure 2D). Both cbMNC- and cbMSC-group animals had significant reduction in contralateral hindlimb misplacement at day 14 after stroke (*P* < 0.05 for MNC and *P* < 0.01 for cbMSC group), relative to PBS group (Figure 2E).

### cbMNCs promote greater reduction in infarct size

**Figure 3:**
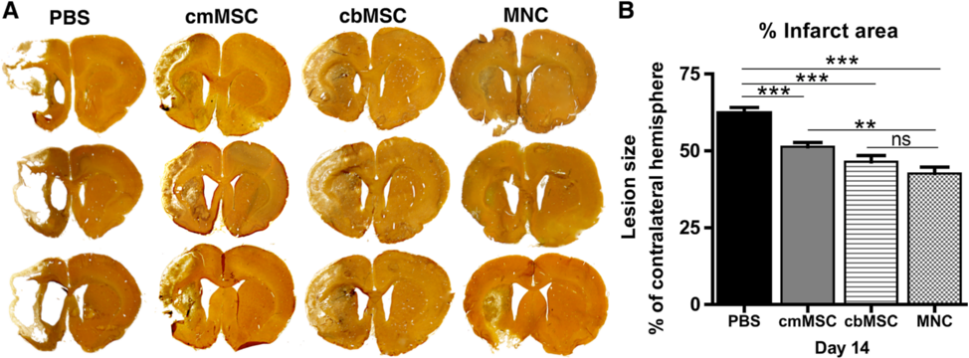
**All three cell types promoted histopathologic recovery, with greater improvement seen in the cbMNC group.** Silver-stained brain sections showed decreased infarct size, reduced ventricular enlargement, and loss of cytoarchitecture in all three cell-treated animals compared with the PBS group **(A)**. Significant reduction in percentage of infarct area across all three cell groups (*P* < 0.001) was seen compared with the PBS group at d 14 after stroke **(B)**. Reduction in lesion size was significantly more in the cbMNC versus cmMSC group (*P <* 0.01). Values are expressed as mean ± SEM. (**) is *P <* 0.01; (***) is *P <* 0.001, and (ns) is nonsignificant.

High-contrast silver staining at day 14 after stroke showed extensive cortical and striatal atrophy, massive loss of cytoarchitecture, and enhanced ventricular enlargement in the PBS group. Transplantation of all three cell types significantly reduced infarct size with apparently reduced ventricular enlargement and loss of cytoarchitecture. The percentage of infarct area, calculated relative to the contralateral hemisphere, was 62.337% ± 5.508% in the PBS group, 42.64% ± 6.56% in the cbMNC group, 46.436% ± 6.3% in the cbMSC group, and 51.337% ± 4.37% in the cmMSC group, (*n* = 10 per group). Although all three cell types were similarly effective in reducing infarct size relative to the PBS group (*P* < 0.001), the reduction in percentage infarct area was significantly greater in the cbMNC group compared with the cmMSC group (*P* < 0.01) (Figure 3B). No significant difference was seen in percentage of infarct area of cbMNC and cbMSC groups or between cbMSC and cmMSC groups.

### cbMNCs, cbMSCs, and cmMSCs can survive in the host ischemic environment and mediate similar immunomodulatory effects

**Figure 4:**
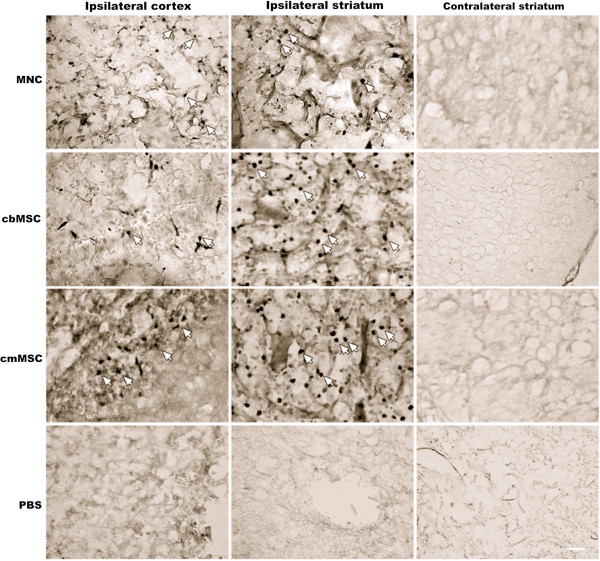
**Distribution of transplanted cells in the ischemic hemisphere relative to the contralateral hemisphere.** All three cell types migrated from the ICA to the ischemic hemisphere and were localized predominantly in the cortex and striatum of the infarct boundary zone (IBZ). Arrows represent MAB1281-positive cells. No MAB1281 (HuNu)-positive cells were found in the contralateral hemisphere of any of the three cell groups. Similarly, no MAB1281 staining was seen in the ipsilateral or contralateral hemisphere of PBS rats. Scale bar = 10 μm.

To identify and characterize the transplanted human cells in the brains of immunocompetent stroke rats, immunohistochemistry was performed on coronal brain sections at 2 weeks after transplantation (*n* = 4 per group). HuNu-positive cells were found in the ipsilateral hemisphere of cbMNC-, cbMSC-, and cmMSC-treated animals. HuNu-positive cells were not present in the contralateral hemisphere of cell-transplanted animals, indicating the propensity of all three cell types to migrate through the ICA and to home sustainably to the region of infarction (Figure 4).

**Figure 5:**
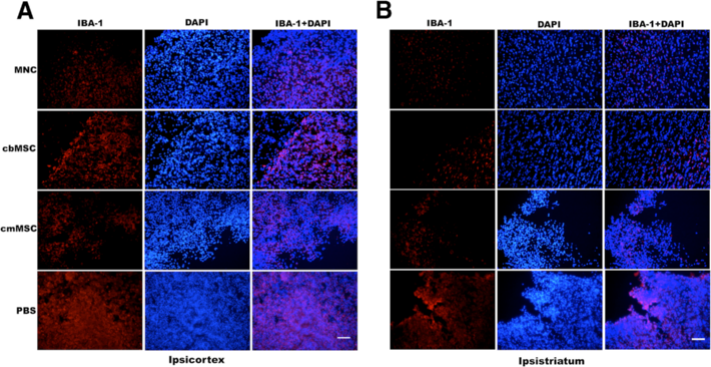
**Photomicrographs showing expression of ionized calcium-binding adapter molecule-1 (Iba-1) in ipsilateral hemisphere of cell-transplanted and PBS-group animals.** A marked decrease in activated macrophage/microglial Iba-1-positive cells was seen in all three cell-group animals compared with the PBS group. The Iba-1-positive cells seen in cell-group animals had both ramified and amoeboidal morphology compared with more-compact amoeboidal morphology seen in the PBS group. Decrease in expression of Iba-1 was more prominent in ipsi-striatum **(B)** than in ipsi-cortex **(A)**. Scale bar = 100 μm.

**Figure 6:**
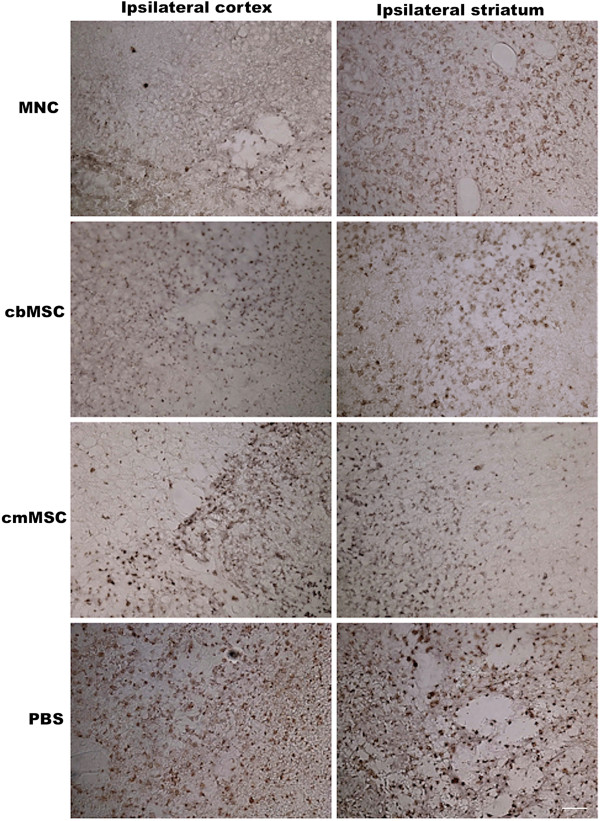
**Representative photomicrographs of CD68-positive cells in the ipsilateral hemisphere of cbMNC-, cb/cmMSC-, and PBS-treated animals.** Animals from all three cell groups showed a trend of fewer CD68^+^-activated microglial cells in both ipsilateral cortex and striatum compared with the PBS-group animals. Scale bar = 10 μm.

Relative to the PBS group, a marked decrease in Iba-1-positive cells (activated macrophages/microglia) was observed in both ipsilateral cortex and more prominently in the striatum of cell-treated animals (Figure 5). Similar to reduction in Iba-1-positive cells, a trend of fewer CD68-positive, activated microglia was seen in all three cell-group animals compared to PBS-group animals (Figure 6).

### Assessment of neuroprotective, antiinflammatory, and antioxidative effects of the three cell types in stroke rats

**Figure 7:**
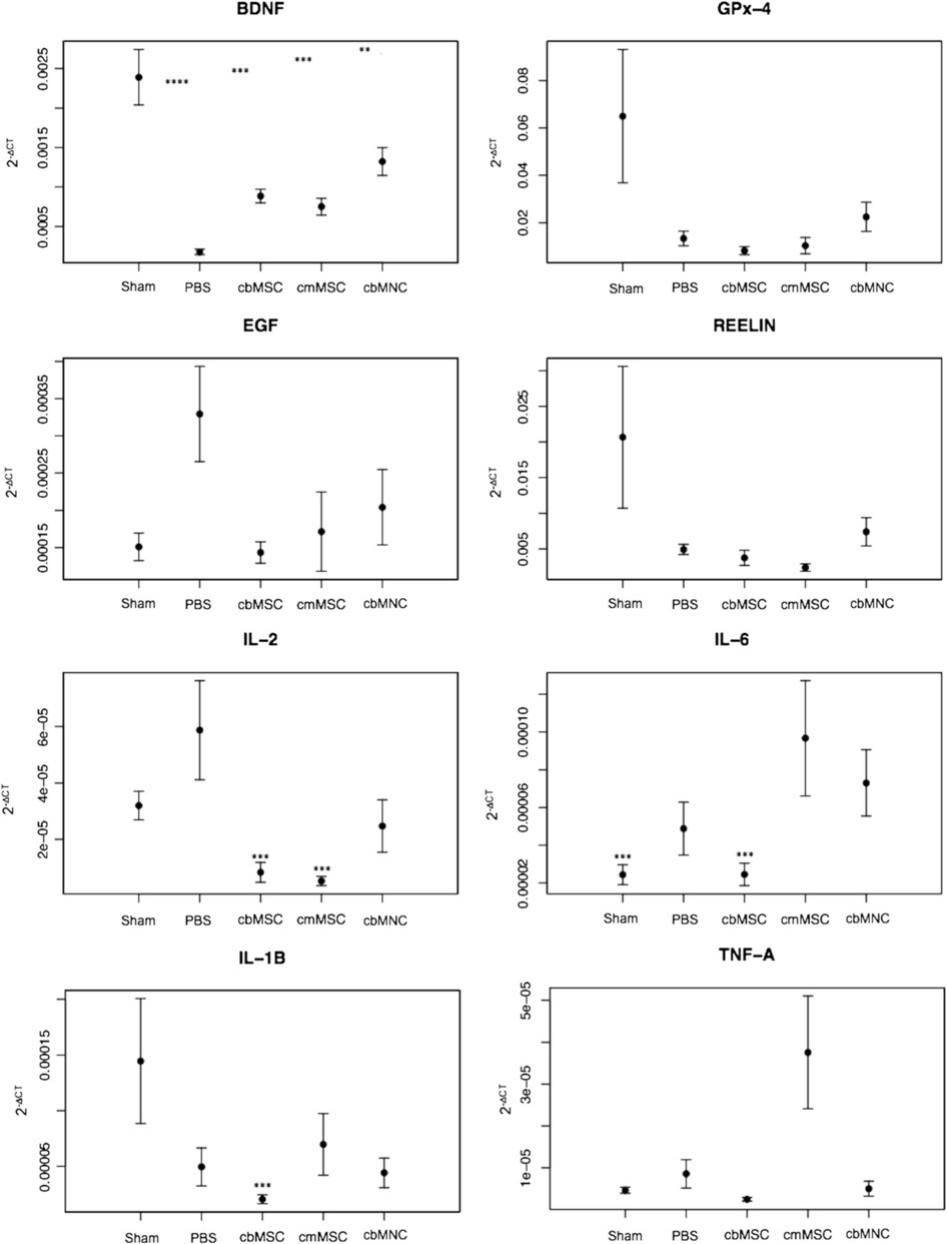
**Quantification of mRNA expression of rat-specific *BDNF, GPx-4, EGF, reelin, IL-2, IL-6, IL-1beta, *and *TNF-alpha in the ipsilateral hemisphere.*** At 2 weeks after stroke, rat *BDNF* mRNA expression in sham rats was significantly more compared with PBS (*P* < 0.0001), cbMNC (*P <* 0.01), and cb/cmMSC (*P <* 0.001) groups. Rat *BDNF* mRNA expression was relatively more in the cbMNC group compared with the cb/cmMSC and PBS groups. No significant difference was seen in expression of rat *EGF*, *reelin*, *GPx-4,* and *TNF-alpha* across the cell-treated, sham, and PBS groups, although there was an enhanced expression of *GPx-4* and *reelin* mRNA in cbMNC and sham animals. Significantly decreased *IL-2* mRNA expression was seen in the cb/cmMSC groups (*P <* 0.001). Compared with the PBS group, expression of *IL-6* mRNA was significantly less in cbMSC- and sham-group animals (*P <* 0.001). Significantly decreased expression of rat *IL-1beta* mRNA was seen only in the cbMSC group (*P < 0.001*). Values are expressed as mean ± SEM, (*) is *P* < 0.05; (**) is *P* < 0.01; (***) is *P* < 0.001; and (****) is *P* < 0.0001.

We assessed the mRNA expression of nine rat-specific genes (*BDNF, GPx-4, EGF, reelin, IL-2, IL-6, IL-10, IL-1beta, TNF-alpha*) and three human-specific genes (*IL-2, IL-6,* and *IL-10*) in the ipsilateral hemisphere of cell-treated, PBS, and sham-group animals. At 2 weeks after stroke, rat *BDNF* mRNA expression was significantly higher in the ipsilateral hemisphere of the sham group compared with the PBS (*P* < 0.0001), cbMNC *(P <* 0.01), and cb/cmMSC (*P <* 0.001) groups. Compared with the cbMSC, cmMSC, and PBS groups, rat *BDNF* mRNA showed a trend of higher expression in the cbMNC group (Figure 7). At day 14 after stroke, a trend of enhanced expression of rat *reelin* and *GPx-4* mRNA was detected in sham and cbMNC-treated animals compared with the PBS group. No significant difference was observed in rat *EGF* mRNA expression among the PBS, sham, and cell-treated groups. Relative to the PBS group, expression of rat *IL-2* mRNA was low in the sham and cell groups, with a significant decrease in the cbMSC and cmMSC groups (*P <* 0.001). Rat *IL-6* mRNA was significantly lower in the cbMSC and sham groups (*P <* 0.001) and was detected at high levels in the cmMSC and cbMNC groups (difference not significant).

The lowest expression of rat *IL-1beta* mRNA was detected in cbMSC-treated animals (*P <* 0.001), with no significant difference between the sham, PBS, cbMNC, and cmMSC groups. No significant difference was detected in expression of rat *TNF-alpha* mRNA between groups, although cmMSCs showed a trend of higher *TNF-alpha* mRNA expression. No expression of rat *IL-10* mRNA or human-origin cytokine mRNA (*IL-2, IL-6, IL-10*) could be detected in any of the study groups.

### cbMNCs and cbMSCs showed no adverse effects and decreased mortality after stroke

No cell-related adverse events were observed in cbMNC- and cbMSC-treated animals after transplantation. Of 23 animals treated with 5 × 10^6^ cmMSCs, one animal developed severe inflammation in the ipsilateral eye within 24 hours of transplantation (see Additional file 4). In contrast, the incidence of severe inflammation of the ipsilateral eye in animals transplanted with 10 × 10^6^ cmMSCs was significantly higher (three of six). Of these four total animals with severe eye inflammation, all three of the animals transplanted with 10 × 10^6^ cells died within 24 hours; however, the animal transplanted with 5 × 10^6^ cmMSCs survived to day 14 (Table 2). A trend of decreased overall mortality after stroke was seen in the cbMNC (8%) and cbMSC (11%) groups relative to the PBS (22%) group. The overall mortality rate was highest in the cmMSC (26%) group, although the difference in mortality of all three cell-group animals and the PBS group was not significant (Table 2). No animals died within 24 hours of cbMNC (10 × 10^6^), cbMSC (5 × 10^6^), or cmMSC (5 × 10^6^) transplantation compared with one animal from the PBS group. In contrast, all six animals transplanted with 10 × 10^6^ cmMSCs died within 24 hours of transplantation (Table 2).

**Table 2:** Adverse effects and mortality after cell transplantation

**Cell type**	**Cell dose**	**Cell-related adverse effects after transplantation**		**% Mortality (number of deaths/total number) after transplantation**	
		**< 24 hours**	**>24 hours**	**<24 hours**	**>24 hours**
cbMNCs	10 × 10^6^	None	None	0 (Nonsignificant)	8% (2/24) (Nonsignificant)
cmMSCs	10 × 10^6^	3/6 animals developed severe inflammation in ipsilateral eye	None	100% (6/6) *P < 0.0001	_
	5 × 10^6^	1/23 animals developed severe inflammation in ipsilateral eye	None	0 (Nonignificant)	26% (6/23) (Nonsignificant)
cbMSCs	5 × 10^6^	None	None	0 (Nonsignificant)	11% (2/18) (Nonsignificant)

Adverse effects and mortality after cell transplantation. The table shows cell-related adverse effects within 24 hours and >24 hours of cell transplantation. Both cbMNC- and cbMSC-group animals had no adverse effects after cell transplantation. One animal transplanted with 5 × 10^6^ cmMSCs and three animals transplanted with 10 × 10^6^ cmMSCs had severe inflammation of the ipsilateral eye within 24 hours of transplantation. The mortality rate was calculated, for two time points, within 24 hours and >24 hours, as a percentage of animals that died to the total animals in each group. A decreasing trend in mortality was seen in the cbMNC and cbMSC groups, but not in the cmMSC group compared with the PBS group. The mortality rate within 24 hours of transplantation in animals transplanted with 10 × 10^6^ cmMSCs was significantly higher compared with the PBS group animals (*P <* 0.0001). *P value relative to the PBS group calculated with the Fisher Exact test. Significance was ascertained at *P <* 0.05.

## Discussion

HUC blood is a promising source of cells for the treatment of stroke, either by using a heterogeneous mix of unprocessed cells or by preselecting a specific cell population from it [11]. Although these two therapeutic avenues have been tested in various preclinical stroke studies with beneficial effects, it is not yet known whether one is more effective than the other. Thus, we compared the cbMNC population, which is a heterogeneous mix of hematopoietic, mesenchymal, and endothelial stem/progenitor cells, along with immature immunological cells [12], with MSCs derived from cbMNCs and cord matrix.

Although, in principle, transplantation of a xenogenic graft might induce a local immune response in the host, preclinical and clinical reports have raised the possibility of using HUC-derived cells without immunosuppression [12,17]. Reports have also suggested that immunosuppression is neuroprotective [73,74]. Therefore, to assess the safety and unbiased efficacy of these cells, we omitted immunosuppression in our study.

Cord-blood-derived cells and other stem cells can be chemotactically attracted to the site of injury [17]. It is thus reasonable to opt for systemic rather than intracerebral delivery to avoid tissue damage at the injection site [75]. Further, selection of intraarterial over intravenous injection was based on study results that showed that more cells reach the MCA territory when transplanted intraarterially [76,77] and another study that showed approximately 96% of cells transplanted intravenously were trapped in the lungs and did not reach the arterial circulation in a traumatic brain injury model [78].

As transplanted cell survival in the host brain of immunocompetent rats was one of our outcome measures, we opted to transplant these cells intraarterially.

A dose-response study of cord-blood-derived cells suggested that delivery of 10^6^ cells was sufficient to result in significant functional recovery and that doses of >10^6^ cells resulted in functional as well as histopathologic recovery [24]. We thus opted for a dose of 10^7^ cells to assess the comparative effects of transplanted cbMNCs and cb/cmMSCs. However, we decreased the dose to 5 × 10^6^ for cb/cmMSCs, as intraarterial injection of 10^7^ cmMSCs resulted in severe inflammation of the ipsilateral eye, followed by acute mortality in our study animals. Thus, 5 × 10^6^ cells was the maximum number of cb/cmMSCs we could safely transplant (Table 2). MSCs have a tendency to aggregate into multicellular globules [79], thus at higher concentrations, they can result in vascular embolization.

Our data suggest that cbMNCs are more effective than cb/cmMSCs in promoting functional recovery and reducing infarct. Although we would have preferred to inject the same doses of cbMNCs and cb/cmMSCs, we think it is unlikely that a twofold difference in transplanted-cell numbers could account for increased beneficial effects of cbMNCs. Furthermore, cbMSCs comprise a very small subset (0.001% to 0.000001%) of cbMNCs [80]. Therefore, it is reasonable to assume that an enriched preselected population of MSCs would have been equally or more effective at a lesser dose, if they were the critical cbMNC subpopulation that mediates recovery. Our flow-cytometry data showed that the cultured fraction of cbMNCs was enriched in CD73^+^CD166^+^CD90^+^CD45^-^HLA-DR^-^ cells (cbMSCs). These cultured cbMSCs showed an approximately 20-fold increase in expression of MSC markers, resulting in a very effective enrichment of cbMSCs from the cbMNCs. Thus, 5 × 10^6^ transplanted cbMSCs contained 10-fold more cbMSCs than in 10 × 10^6^ cbMNCs. The enhanced recovery in the cbMNC group compared with the enriched cb/cmMSC groups could be due to additional cell fractions in the cbMNCs that might secrete factors important for recovery. Thus, the as-yet-uncharacterized functional properties of cbMNC subpopulations and their possible interactions, rather than total cell numbers, could be responsible for the beneficial effects of transplanted cbMNCs. The enhanced recovery with cbMNCs thus may be partially attributable to the integrative effects of various stem/progenitor cell fractions present in this cell population.

A recent study [81] compared the effects of cbMNCs with CD34-enriched and CD34-depleted cbMNC fractions in spontaneously hypertensive stroke rats. The study reported superior effects of cbMNCs relative to the CD34^+^ and CD34^-^ fractions, suggesting the possibility that the combined effect of other cell fractions was necessary for the overall neuroprotective effect.

Although rats in both the cbMNC and cbMSC groups showed improvement in sensory motor functions, only cbMNC-treated rats showed early (within 7 days) recovery in the grid-walk test. This was likely attributable to the significant improvement seen in both contralateral forelimb and hindlimb deficits in this group. Infarct size was also most reduced in the cbMNC group and was significantly smaller than that in the cmMSC group. Although cbMNCs have been shown to produce various growth factors, such as *VEGF* and *BDNF*[82], it is probably their ability to induce enhanced expression of endogenous *BDNF*[29] that partially resulted in enhanced recovery in this group. *BDNF* is known to mediate proliferation of existing vascular endothelial cells [83,84], survival and migration of neuronal cells, along with modulation of their synaptic functions [51,52], and to exert neuroprotective effects via downregulation of neuronal NOS (nNOS) activity [85]. Intravenous infusion of *BDNF* has been shown to reduce infarct volume as early as 5 hours after stroke [47].

Further, the increased mRNA expression of rat-specific *BDNF* seen in sham versus PBS animals implies a reduction in the *BDNF* endogenous levels after stroke. Thus, cbMNCs and, to a lesser extent, cb/cmMSCs, possibly restore the stroke-induced depletion of endogenous *BDNF*[86]. In addition, the trend of increased expression of rat *GPx-4* mRNA in cbMNC-treated animals indicates a possible role of MNCs in abating the effects of oxidative stress on *GPx-4* levels. The restored *GPx-4* might in turn downregulate lipid peroxidation, resulting in decreased neuronal cell death and enhanced overall recovery.

Similar to earlier reports [16,24], transplanted cbMNCs, cbMSCs, and cmMSCs were seen predominantly in the ischemic hemisphere where homing is likely facilitated by chemokine receptor type-4 (CXCR4)-CXCL12 or CD117-stem cell factor (SCF) interactions. Upregulation of CXCL12/stromal-derived factor 1(SDF-1) has been reported in the ischemic penumbra [87,88], and its interaction with (CXCR-4)/CD184 (expressed by all three cell populations used in our study) is known to promote migration of cbMNCs [89]. Also, enhanced expression of SCF has been reported in neurons within the injured hemisphere [90] and might have played a role in directed migration of transplanted cb/cmMSCs, both of which had significant expression of the SCF receptor CD117, as shown in our comparative flow-cytometry experiment.

We observed decreased numbers of CD68- and amoeboidal Iba-1-positive cells in our cell-treated versus PBS animals, indicating a decrease in activated microglia. These resident immune cells are known to acquire a phagocytic phenotype after stroke that disrupts the blood–brain barrier and increases inflammation through release of proinflammatory cytokines, free radicals, and recruitment of leukocytes from the circulatory system [91,92]. Thus, a decrease in activated microglial cells in the injured brain after HUC-derived cell treatment represents an immunomodulatory effect that could reduce neuroinflammation and increase recovery. Although how HUC-derived cells suppress activated microglia is unknown, a recent study implicates the role of CD11b^+^ and CD19^+^ cbMNCs in reducing microglial survival and CD4^+^ in sustaining microglia *in vitro* after hypoxic conditions [91]. In our study, cbMNCs were CD19^+^CD4^+^, cmMSCs were CD19^-^, but had enriched CD4^+^ expression, whereas cbMSCs had negligible expression of both CD19 and CD4. Thus, markedly reduced activated microglial cells in each of these three cell groups indicates that additional factors could be involved in mediating microglial suppression as well as the possibility that CD19^+^ cells override the protective effects of CD4^+^ cells.

In stroke, various pro-inflammatory cytokines are secreted from infiltrated leukocytes and macrophages through activation of resident microglia. The insignificant differences in *IL-6, IL-β, and TNF-alpha* mRNA expression within the cbMNCs and cmMSCs groups compared with control animals may be due to time-dependent expression patterns. These cytokines have been shown to peak in expression in the ischemic hemisphere at day 7 after stroke, which was decreased by day 14 [93]. The significant decrease in *IL-2, IL-6,* and *IL-1beta* mRNA in the cbMSC group could indicate a mechanistic shift of action of these cells from their parental cbMNC population. Whereas suppression of proinflammatory factors might mediate the recovery observed in cbMSC-treated animals, in the cbMNC group, recovery is more likely associated with the release of growth factors and the ability to attenuate oxidative stress. The negligible antiinflammatory cytokine *IL-10* mRNA seen in our study is similar to that of previous reports [93] in which no expression was seen at day 14 after stroke.

The absence of any human cytokine expression in the ipsilateral hemisphere of the transplanted animals is possibly due to the moderate number of surviving human cells, low levels of cytokine secretion by the transplanted cells, or reduced overall inflammation by day 14 after stroke. Future studies assessing the treatment and time-dependent profile of these cytokines will enhance our understanding of inflammation and the effects of umbilical cord cell subtypes on the same. At 2 weeks after stroke, insignificant differences in the expression of *reelin* and *EGF* mRNA between the PBS, sham, and cell groups could indicate that *reelin*-mediated migration of progenitors and *EGF*-mediated recovery after stroke is time dependent.

Cell survival in immunocompetent stroke rat brains and the absence of adverse events in cbMNCs and cbMSCs might be due to their immunomodulatory function in addition to the presence of phenotypically and functionally immature T-lymphocytes [94]. Cord blood has a higher percentage of homogeneous regulatory T-lymphocytes (Tregs) compared with heterogeneous Tregs present in peripheral blood and a smaller percentage in bone marrow [95]. Tregs are known to dampen the immune response [96] and have been shown to exert potent antiinflammatory neuroprotective effects after stroke [97]. HUC blood Tregs have also been shown clinically to prevent allogeneic acute graft-versus-host disease (GVHD) [98]. It is therefore important to understand the role of this subpopulation in mediating the survival of transplanted HUC-derived cells without adverse effects and whether these cells play a role in poststroke recovery. Although many studies with Treg-depleted donor allografts have shown enhanced GVHD [99-101], it is worth exploring whether Treg-depleted cord-blood-derived cells home and exert similar neuroprotective effects in stroke, as seen in this study. Further, it would be interesting to determine whether cord-blood and matrix cells have phenotypically and functionally similar Treg populations.

Last, although both cbMSCs and cmMSCs showed similar expression of MSC-specific markers, they differed in their expression of Lin1, CD56, CD4, and CD14, which could account for the differences in functional and histopathologic recovery seen with these cells. It would be interesting to determine whether these differences are preserved with cbMSCs and cmMSCs isolated from the same donor. Further, because of phenotypical and functional heterogeneity within MSC preparations and variations between MSC donors, it would be informative to include multiple MSC preparations from multiple donors in MSC studies [102,103].

We analyzed cbMSCs isolated from a single donor, which is a limitation of our study. Future studies comparing cbMSCs isolated from multiple donors would therefore provide valuable information on donor-dependent and/or MSC-subpopulation variations in the context of cbMSC transplantation after stroke.

## Conclusions

Our study data show that cbMNCs, cbMSCs, and cmMSCs are effective in ameliorating the effects of stroke. However, cbMSCs, an enriched cell population of cbMNCs, or cmMSCs, did not show superior performance in stroke rats compared with the heterogeneous cbMNCs. cbMNCs are widely accessible and more easily prepared compared with processed and *ex vivo* expanded MSCs. Further, the sustainable homing of cbMNCs similar to cb/cmMSCs and the absence of severe cell-related adverse effects, as seen with cmMSCs, suggests cbMNCs to be a promising cell therapy for stroke.

## Abbreviations

BDNF: Brain-derived neurotrophic factor; cbMSCs: cord blood mesenchymal stromal cells; CCA: common carotid artery; cmMSCs: cord matrix MSCs; CXCR-4: chemokine receptor type; ECA: external carotid artery; FACS: fluorescence-activated cell sorting; GAPDH: glyceraldehyde-3-phosphate dehydrogenase; GPx-4: glutathione peroxidase-4; HUC: human umbilical cord; HUCB: human umbilical cord blood; HuNu: human nuclei; Iba-1: ionized-calcium-binding adapter molecule; IBZ: infarct boundary zone; ICA: internal carotid artery; MCAo: middle cerebral artery occlusion; MNCs: cord blood mononuclear cells; SCF: stem cell factor; SDF-1: stromal-derived factor-1; VSELs: very small embryonic-like stem cells.

## Competing interests

The authors have no competing interests to declare.

## Authors’ contributions

NK conceived the study, designed and executed experiments, collected, assembled, analyzed and interpreted data, and wrote and edited the manuscript. NCM contributed to qPCR experiments, analysis of data, and revision of the manuscript. KP contributed to analysis and interpretation of data and was involved in manuscript writing and editing. RS participated in analysis and interpretation of data, and contributed in writing and editing of the manuscript. GKS was involved in experimental design, analysis and interpretation of data, and writing and editing of the manuscript. All authors have read and approved the final manuscript.

## Supplementary Material

Additional file 1 **Description of Data.** List of antibodies used in multicolor flow cytometry. Twenty-seven phenotypic cell markers were used for comparative immunophenotypic characterization of cbMSCs, cmMSCs, and cbMNCs. Antibodies for 27 markers were divided into six FACS tubes according to their conjugated fluorochromes and their emission range. Click here for file

Additional file 2 **Description of Data.** Representative flow-cytometry dot plots of cbMSCs, cmMSCs, and cbMNCs. The plots illustrate expression of phenotypic cell markers CD59, CD184, CD106, CD90, CD210, CD73, CD56, CD44, CD117, CD123, CD166, HLA-ABC, HLA-DR. In each plot, percentage of cells positive for a given marker is shown on right, and percentage of cells negative for the same marker is shown on the left. Gates were set according to the unstained controls and compensation was done by single-color-stained BD-CompBeads. Click here for file

Additional file 3 **Description of Data.** Representative flow-cytometry dot plots of cbMSCs, cmMSCs, and cbMNCs. The plots illustrate expression of phenotypic cell markers CD3, CD4, CD8, CD7, CD14, CD25, CD14, CD45, CD34, CD133, CD33, CD19, and CD106 (B) in all three cell groups (cbMSCs, cmMSCs, and cbMNCs). In each plot, percentage of cells positive for a given marker is shown on the right, and percentage of cells negative for the same marker is shown on the left. Gates were set according to the unstained controls, and compensation was done by single-color-stained BD-CompBeads. Click here for file

Additional file 4 **Description of Data.** Photographs of two stroke rats taken 72 hours after cmMSC transplantation. One stroke rat from cmMSC group had severe inflammation in ipsilateral eye post cell (5 × 10^6^) transplantation, which persisted until 14 days (A). The eye can be well demarcated from normal ipsilateral eye of another cmMSC-transplanted animal with no adverse effect (B). Similar inflammation of the ipsilateral eye was also seen in three animals transplanted with 10 × 10^6^ cmMSCs, all of which died within 24 hours of transplantation. Click here for file
