# Editorial Expression of Concern: Intraarterial transplantation of human umbilical cord blood mononuclear cells is more efficacious and safer compared with umbilical cord mesenchymal stromal cells in a rodent stroke model

**DOI:** 10.1186/s13287-024-03661-z

**Published:** 2024-02-16

**Editorial Expression of Concern: Stem Cell Research & Therapy 2014, 5:45** 10.1186/scrt434

The Editors-in-Chief are issuing this editorial expression of concern to alert readers that in Figure 4 there is overlap between the cbMSC and the cmMSC images of the Ipsilateral Striatum. Due to the age of the article the authors are unable to access and provide the original raw data for evaluation.

All authors agree to this editorial expression of concern.

