# Supplementary figures and images for: Intraarterial transplantation of human umbilical cord blood mononuclear cells is more efficacious and safer compared with umbilical cord mesenchymal stromal cells in a rodent stroke model
Source: Stem Cell Res Ther. 2014 Apr 1;5(2):45. doi: 10.1186/scrt434 (PMC4055161; doi:10.1186/scrt434)

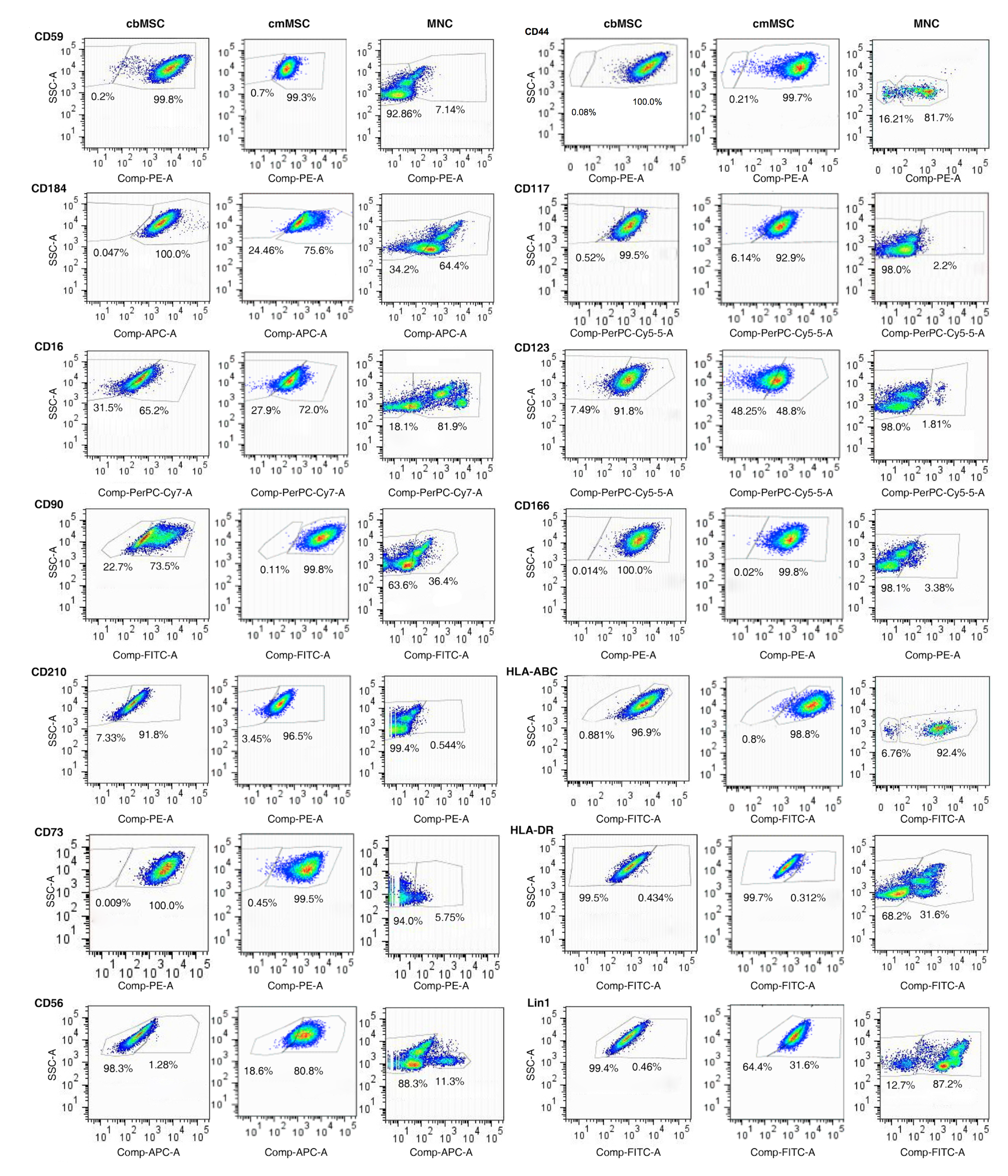

Supplement: Additional file 1 — Description of Data. List of antibodies used in multicolor flow cytometry. Twenty-seven phenotypic cell markers were used for comparative immunophenotypic characterization of cbMSCs, cmMSCs, and cbMNCs. Antibodies for 27 markers were divided into six FACS tubes according to their conjugated fluorochromes and their emission range. [file scrt434-S1.jpeg]

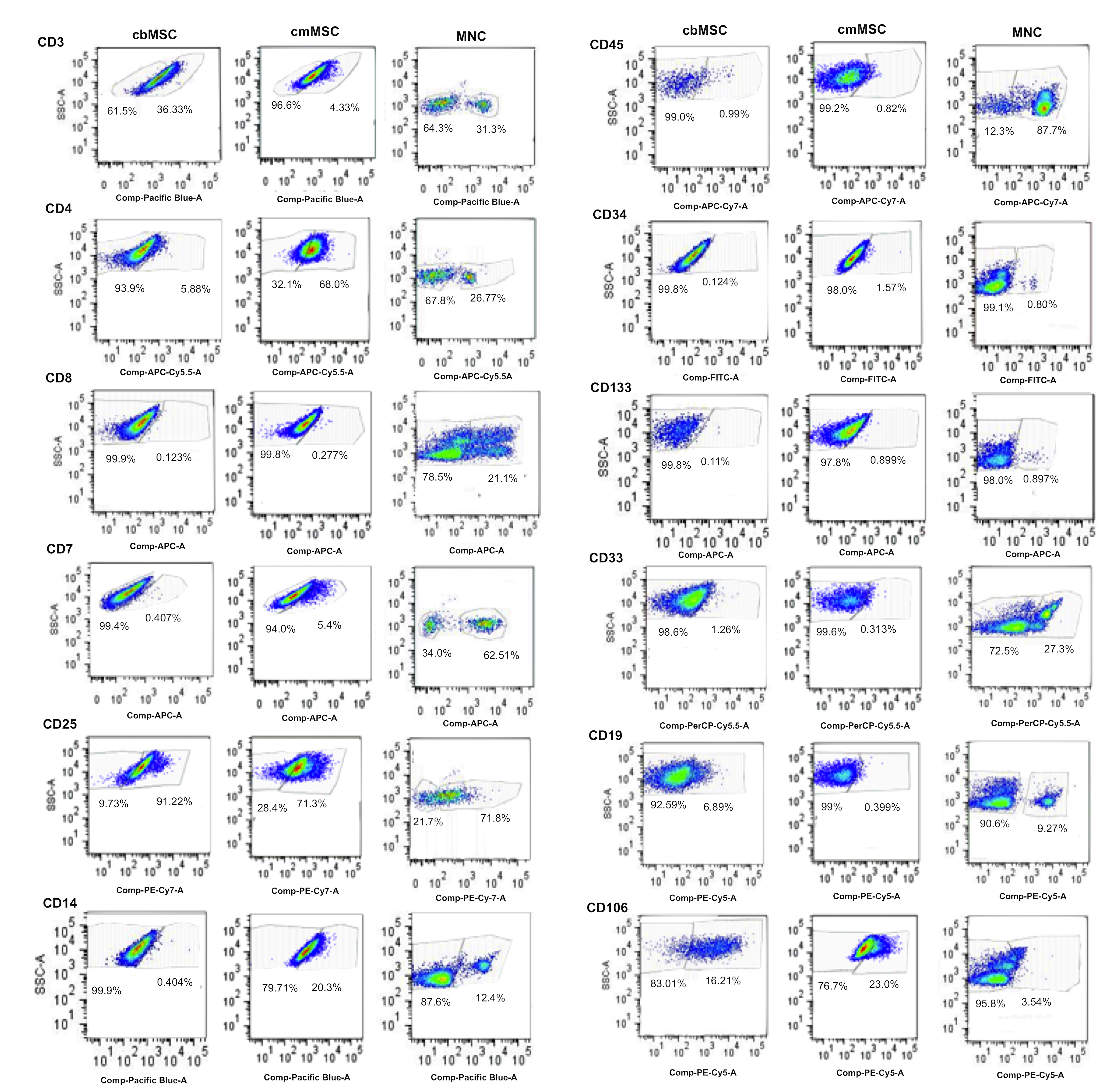

Supplement: Additional file 2 — Description of Data. Representative flow-cytometry dot plots of cbMSCs, cmMSCs, and cbMNCs. The plots illustrate expression of phenotypic cell markers CD59, CD184, CD106, CD90, CD210, CD73, CD56, CD44, CD117, CD123, CD166, HLA-ABC, HLA-DR. In each plot, percentage of cells positive for a given marker is shown on right, and percentage of cells negative for the same marker is shown on the left. Gates were set according to the unstained controls and compensation was done by single-color-stained BD-CompBeads. [file scrt434-S2.jpeg]

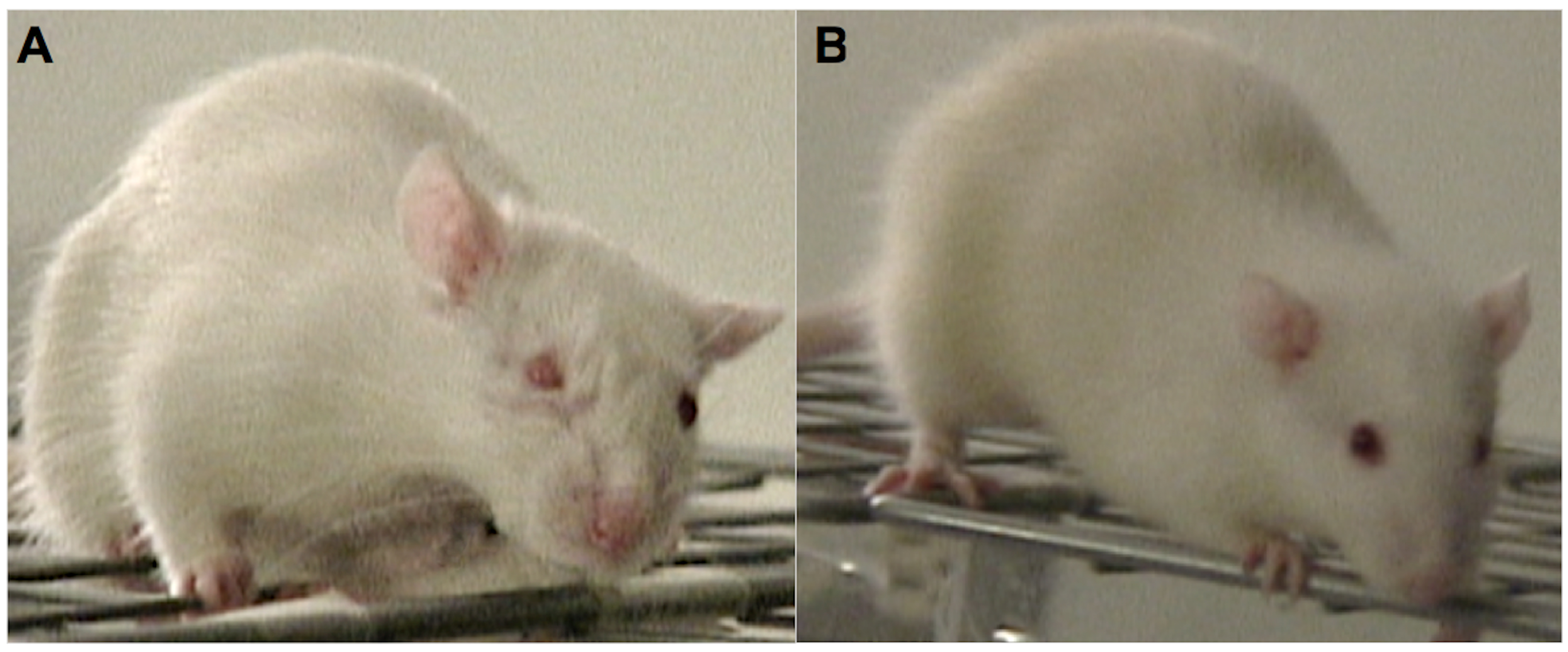

Supplement: Additional file 3 — Description of Data. Representative flow-cytometry dot plots of cbMSCs, cmMSCs, and cbMNCs. The plots illustrate expression of phenotypic cell markers CD3, CD4, CD8, CD7, CD14, CD25, CD14, CD45, CD34, CD133, CD33, CD19, and CD106 (B) in all three cell groups (cbMSCs, cmMSCs, and cbMNCs). In each plot, percentage of cells positive for a given marker is shown on the right, and percentage of cells negative for the same marker is shown on the left. Gates were set according to the unstained controls, and compensation was done by single-color-stained BD-CompBeads. [file scrt434-S3.jpeg]
