# Supplementary material for: Intraarterial transplantation of human umbilical cord blood mononuclear cells is more efficacious and safer compared with umbilical cord mesenchymal stromal cells in a rodent stroke model
Source: Stem Cell Res Ther. 2014 Apr 1;5(2):45. doi: 10.1186/scrt434 (PMC4055161; doi:10.1186/scrt434)
Supplement: Additional file 4 — Description of Data. Photographs of two stroke rats taken 72 hours after cmMSC transplantation. One stroke rat from cmMSC group had severe inflammation in ipsilateral eye post cell (5 × 10^6^) transplantation, which persisted until 14 days (A). The eye can be well demarcated from normal ipsilateral eye of another cmMSC-transplanted animal with no adverse effect (B). Similar inflammation of the ipsilateral eye was also seen in three animals transplanted with 10 × 106 cmMSCs, all of which died within 24 hours of transplantation. [file scrt434-S4.doc]

Additional file 1

| Antibodies (label) | Distributor # Cat.No. | Volume (μl) | Tube number |
| --- | --- | --- | --- |
| 7 AAD | BD Pharm.#559925 | 5 | 1, 2, 3,4, 5, 6 |
| CD7 (APC) | Biolegend #343107 | 20 | 1 |
| CD4 (Alexafluor 700) | BD Pharm.#557922 | 5 | 1, 2 |
| CD25 (PE-Cy7) | BD Pharm.#557741 | 5 | 1, 2 |
| CD3 (PB) | Biolegend.#300329 | 20 | 1 |
| CD44 (PE) | BD Pharm.#555479 | 20 | 1 |
| HLA-ABC (FITC) | BD Pharm.#555552 | 20 | 1 |
| CD14 (PB) | BD Biosciences # 558121 | 5 | 2, 3 |
| CD90 (FITC) | BD Biosciences. # 555595 | 20 | 2 |
| CD56 (APC) | BD Pharm.555518 | 20 | 2 |
| CD210 (PE) | Biolegend#308803 | 20 | 2 |
| CD45 (APC-Cy7) | BD Biosciences #557833 | 5 | 3, 4 |
| CD34 (FITC) | BD Biosciences# 555821 | 20 | 3 |
| CD133 (APC) | Miltenyi Biotec.#130-090 | 10 | 3 |
| CD117 (PerCP-Cy5.5) | Biolegend#313213 | 5 | 3 |
| CD73 (PE) | BD Pharmingen#561014 | 20 | 3 |
| CD16 (PE-Cy7) | Biolegend#302015 | 20 | 4 |
| CD166 (PE) | Biolegend #343903 | 20 | 4 |
| Lineage 1 (FITC) | BD Biosciences#340546 | 20 | 4 |
| CD123 (PerCP-CY5.5) | BD Pharm.#558714 | 20 | 4 |
| CD59 (PE) | Biolegend #304707 | 20 | 5 |
| CD106 (PE-Cy5) | Biolegend # 305808 | 20 | 5 |
| CD184 (APC) | Biolegend #306509 | 20 | 5 |
| HLA-DR (FITC) | BDPharm.#556643 | 20 | 5 |
| CD19 (FITC) | BD Pharm.#555412 | 20 | 6 |
| CD8 (Alexa Fluor® 647) | BD Pharm.#557708 | 5 | 6 |
| CD33 (PerCP-Cy5.5) | Biolegend #303413 | 5 | 6 |
